# Accelerated biological aging mediates the association between periodontal disease and cognitive function in older adults

**DOI:** 10.1093/geroni/igaf086

**Published:** 2025-08-07

**Authors:** Xiang Qi, Huabin Luo, Zhijing Xu, Ruotong Liu, Bei Wu

**Affiliations:** Rory Meyers College of Nursing, New York University, New York, New York, United States; Brody School of Medicine, East Carolina University, Greenville, North Carolina, United States; Rory Meyers College of Nursing, New York University, New York, New York, United States; Rory Meyers College of Nursing, New York University, New York, New York, United States; Rory Meyers College of Nursing, New York University, New York, New York, United States

**Keywords:** Oral health, Dentistry, Alzheimer’s disease, Dementia, Geroscience

## Abstract

**Background and Objectives:**

Periodontitis is a prevalent chronic inflammatory gum disease in older adults and has been linked to cognitive decline, but underlying mechanisms are unclear. The geroscience hypothesis provides a framework for this link, positing that fundamental aging processes (eg, chronic inflammation) drive multiple age-related diseases. We aimed to determine whether accelerated biological aging mediates the association between periodontal disease and cognitive function in older adults.

**Research Design and Methods:**

Data were analyzed from 1950 adults aged ≥60 years in the National Health and Nutrition Examination Survey 1999–2002. Periodontal status was clinically assessed (mean clinical attachment loss [CAL], periodontal probing depth [PD], and periodontitis defined by CDC/AAP thresholds). Cognitive performance was measured with the Digit Symbol Substitution Test (DSST). Biological aging was quantified using Klemera-Doubal method (KDM) and Phenotypic Age (PhenoAge) algorithms. Multivariable linear regressions and mediation analyses (adjusted for sociodemographic, behavioral, and health factors) were conducted to evaluate associations and the proportion of the periodontal-cognition link mediated by biological age acceleration (BAA).

**Results:**

Periodontitis was significantly associated with poorer cognitive function (DSST standardized β=−0.095, *P* < .01) and higher KDM-BAA (β = 0.812, *P* < .001) and PhenoAge Acceleration (β = 1.004, *P* < .001). Each 1-mm increase in CAL was associated with lower DSST scores (β=−0.048, *P* < .01), greater KDM-BAA (β  =  0.221, *P* = .031), and higher PhenoAge Acceleration (β = 0.475, *P* < .001). Higher BAA was independently associated with lower cognitive scores (KDM-BAA β=−0.009, *P* = .021; PhenoAge Acceleration β=−0.008, *P* = .003). Mediation analyses showed KDM-BAA and PhenoAge Acceleration mediated approximately 5.7%–15.1% (all indirect effects *P* < .05) of the total periodontal-cognition relationship.

**Discussion and Implications:**

Accelerated biological aging partially mediates the relationship between periodontal disease and cognitive function, supporting a novel geroscience-based mechanism linking oral inflammation and cognitive decline. Future interventions targeting oral health could simultaneously mitigate systemic aging and protect cognitive function.

Translational Significance Periodontal disease in older adults is associated with cognitive decline, but underlying mechanisms remain unclear. Accelerated biological aging partially mediates this link, highlighting a key oral-systemic aging pathway. This insight suggests innovative interventions at multiple levels: at the individual level, better oral hygiene could preserve cognitive health; at the organizational level, healthcare systems might integrate oral care into cognitive decline prevention; and at the societal level, public health strategies targeting oral-systemic aging processes could potentially reduce the cognitive impairment burden and promote healthy brain aging.

## Introduction

As the population ages, more individuals face late-life ­cognitive decline and dementia. Therefore, identifying modifiable ­factors to promote healthy brain aging is crucial.^1^ Oral health may be one such factor, particularly periodontitis—a chronic inflammatory gum disease that affects about 75% of adults in the United States aged ≥65 years and is a leading cause of tooth loss.^2^ Beyond its local destructive effects, periodontitis contri­butes to systemic inflammation^3^ and is linked to adverse health outcomes.^4–8^ Moreover, studies show older adults with periodontitis perform worse on cognitive tests and have a higher risk of cognitive impairment or dementia than those with healthy gums,^3^^,^^9–12^ though the underlying mechanisms remain unclear.

The geroscience hypothesis provides a useful framework for this oral-systemic connection. It posits that fundamental biological aging processes (eg, chronic inflammation, cellular senescence, and mitochondrial dysfunction) drive many age-related diseases.^13–15^ Accordingly, conditions as diverse as cardiovascular disease, diabetes, and neurodegenerative disorders may share overlapping etiologies rooted in the biology of aging. Chronic periodontitis exemplifies this idea: it becomes more common with age and may accelerate systemic aging processes that contribute to other chronic conditions.^15^^,^^16^ Notably, chronological age is an imperfect health indicator because people biologically age at different rates.^13^^,^^14^ Recent advances allow “biological age” to be measured via composite biomarkers that better reflect an individual’s functional age than years lived. Such blood-chemistry biological age estimators are associated with physical decline, multimorbidity, and mortality risk.^17–21^ Importantly, periodontitis has been linked to features of accelerated aging: mitochondrial dysfunction,^22^ systematic inflammation,^3^^,^^6^ and shortened telomeres,^23^ all are considered hallmarks of aging.^14^ These findings suggest periodontitis might hasten the aging trajectory, heightening “gerovulnerability” (susceptibility to age-related dysfunction) in affected adults.^24^ In the context of cognitive health, this raises the question: could chronic periodontal disease impair cognition by accelerating biological aging?

To date, however, the potential mediating role of accelerated biological aging in the link between periodontitis and cognitive decline remains understudied. Most research has investigated the oral-cognitive connection or the biology of aging in isolation, without integrating or linking these domains.^15^^,^^16^^,^^25^ To address this gap, the present study investigates whether biological age acceleration (BAA) mediates the association between periodontal condition and cognitive function in older adults. We analyzed data from a nationally representative sample of adults in the United States aged ≥60, which included clinical periodontal examinations, blood-based biological age measures, and cognitive testing. We also examined whether associations varied by periodontitis severity, anticipating a dose-response (more severe disease showing stronger links to accelerated aging and cognitive impairment). We hypothesized that periodontal conditions (presence of periodontitis, or greater clinical attachment loss [CAL] and probing depth [PD]) would be associated with higher BAA and lower cognitive performance, and that BAA would mediate the periodontitis-cognition association.

## Methods

### Study design and sample

This cross-sectional analysis used data from the 1999–2000 and 2001–2002 cycles of the National Health and Nutrition Examination Survey (NHANES), a complex stratified survey of the U.S. population. In these cycles, NHANES included cognitive testing for adults aged ≥60 and measured key biomarkers (eg, C-reactive protein) required for biological age calculations (later NHANES cycles with cognitive testing did not include these biomarkers). Data were collected via household interviews and clinical examinations in mobile centers.^26^ Detailed information on survey design and data collection can be found elsewhere.^26^ We combined the two cycles (with 4-year sample weights) to maximize sample size. The analytic sample included adults aged ≥60 with at least one natural tooth who completed the cognitive assessment, oral health examination, and laboratory blood tests. After excluding those with missing data in these measures (<5% of eligible participants), the final sample was 1950. All participants provided written informed consent, and the NHANES protocol was approved by the NCHS Institutional Review Board. A flowchart of sample selection is shown in Figure S1 (see online supplementary material for a color version of this figure).

### Measures

#### Periodontal assessment

In NHANES 1999–2000, CAL and PD were measured at two sites (mid-buccal and mesio-buccal) per tooth in two randomly selected quadrants (one upper, one lower).^27^ In 2001–2002, the exam expanded to three sites per tooth (adding disto-buccal) in all quadrants. For consistency, we used only the two common sites in both protocols. Third molars were excluded because of their frequent extraction in young adulthood.^27^ Periodontitis case definitions were applied based on Centers for Disease Control/American Academy of Periodontology (CDC/AAP) guidelines for population surveillance.^28^^,^^29^ Specifically, mild periodontitis was defined as ≥2 interproximal sites with CAL ≥3 mm, and ≥2 interproximal sites with PD ≥4 mm (not on same tooth) or one site with PD ≥5 mm; moderate periodontitis was defined as the presence of ≥2 interproximal sites with CAL ≥4 mm (not on the same tooth) or ≥2 interproximal sites with PD ≥5 mm (not on the same tooth); and severe periodontitis was defined as ≥2 interproximal sites with CAL ≥6 mm (not on the same tooth) and ≥1 interproximal site with PD ≥5 mm (see Table S1 for details).^4^^,^^28^^,^^29^ Participants meeting either moderate or severe criteria were classified as having periodontitis in primary analyses, and those with neither (including mild or no disease) were classified as having no periodontitis.^4^^,^^28^^,^^29^ In addition to this categorical classification, we also evaluated the periodontal conditions using mean PD (mm, averaged across measured sites) and mean CAL (mm, averaged across measured sites) to minimize the bias due to the different clinical measures of periodontitis between the two NHANES sampling periods.^3^^,^^22^ For secondary analyses, we examined periodontitis severity as a three-category variable (no/mild, moderate, severe).

#### Biological age estimation

We estimated each participant’s biological age using two validated algorithms: the Klemera–Doubal method (KDM Biological Age) and Phenotypic Age (PhenoAge).^19^^,^^20^ Both integrate multiple clinical and biochemical markers to yield an age in years reflecting physiological status. We followed published procedures and used NHANES III training coefficients for these algorithms.^19^^,^^20^ Briefly, KDM Biological Age was calculated from 14 biomarkers (12 blood chemistry measures, systolic blood pressure, and lung function [forced expiratory volume]) using the KDM.^19^ The PhenoAge algorithm, developed by Levine et al., uses nine clinical biomarkers combined in a weighted index calibrated to mortality risk.^20^ The selected markers, algorithms, and corresponding R code can be found in the R package named “BioAge” at: https://github.com/dayoonkwon/BioAge.^30^ The details in both algorithms for calculating BAA are also shown in Supplementary Methods. For each participant, we then derived BAA as the residual from regressing biological age on chronological age. This residual represents the portion of biological age unexplained by chronological age. A positive residual (BAA > 0) indicates the individual’s body is older than expected (accelerated aging), while a negative residual (BAA < 0) indicates a biologically younger profile.^17^^,^^20^ We calculated two BAA measures: KDMAge Acceleration and PhenoAge Acceleration, corresponding to the two biological age algorithms.

#### Cognitive function assessment

Cognitive function was assessed with the DSST, a neuropsychological test from the Wechsler Adult Intelligence Scale.^31^ The DSST primarily measures processing speed and executive function, and performance also involves attention, visuomotor coordination, and working memory—domains sensitive to aging.^32^ Given that DSST is a single test focused on specific domains, we interpret lower DSST scores as reflecting poorer cognitive function in those domains, but we caution that it is not a comprehensive measure of dementia or overall cognitive ability. In NHANES, participants were given a sheet with a digit-symbol key and had 2 minutes to write the corresponding symbols for as many digits as possible. The score is the number of correct matches (higher scores = better function), with a maximum of 133. We treated the DSST score as a continuous outcome.

#### Covariates

We included a range of covariates based on known associations of oral health with cognitive health.^33–35^ Sociodemographic covariates included age, sex, race/ethnicity (Non-Hispanic White, Non-Hispanic Black, Hispanic, or other), education (< high school, high school, some college, or ≥ bachelor’s degree), and annual household income (<$20 000, $20 000–$74 999, or ≥$75 000). Health behaviors included smoking status (never; former [≥100 lifetime cigarettes, not current]; current) and alcohol use (≥12 drinks in the past year vs. fewer). We also adjusted for dental care utilization (time since last dental visit: <1 year, 1–3 years, >3 years). Health status covariates included body mass index (BMI) and chronic conditions (hypertension, diabetes, arthritis, dyslipidemia, and cardiovascular disease), defined by self-report or clinical examination in mobile centers (see Table S2). We considered other comorbidities (eg, history of stroke, liver disease, and cancer), but these were not retained in final models as they did not materially affect associations. All primary analyses were adjusted for the core covariates listed above.

#### Statistical analyses

All analyses accounted for NHANES sample weights and complex survey design. First, we described the sample characteristics by periodontal status. Participants were stratified by periodontitis severity (no, mild, moderate, severe), and we compared groups using *t*-tests or analysis of variance (ANOVA) for continuous variables and χ^2^ tests for categorical variables (nonparametric tests for skewed variables). All analyses were conducted using R Studio 4.2 and STATA MP 18.0 (StataCorp). Statistical significance was defined as a two-tailed *P* < .05.

Next, we used multivariable linear regression to examine associations between periodontal status (exposures) and outcomes (BAA measures and cognitive score). Periodontal condition was operationalized three ways: (1) periodontitis (no, mild, moderate, and severe, per the case definition above); (2) mean CAL (mm); and (3) mean PD (mm). For each exposure, we ran four models with sequential adjustments: Model 1 (unadjusted); Model 2 (adjusted for sociodemographics: age, sex, race/ethnicity, education, income); Model 3 (additionally adjusted for health behaviors: smoking, alcohol, dental care utilization); and Model 4 (further adjusted for health status: BMI and chronic conditions). From these models, we obtained survey-weighted β coefficients and standard errors (SE) for each association (periodontal measure with cognitive score, KDMAge Acceleration, and PhenoAge Acceleration), representing the difference in outcome per unit difference in exposure (or relative to the reference category for categorical exposures). The summary of all analytical models and variables included is presented in Table S3.

**Figure 1.**
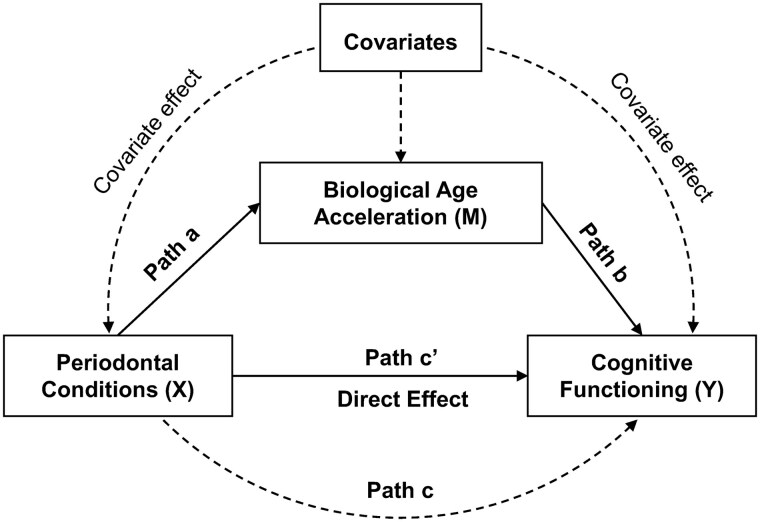
Theoretical diagram of the mediation model for the association between periodontal condition (exposure) and cognitive functioning (outcome), with the BAA as a mediator. Path a: regress “mediator (M)” on “exposure (X)” to examine if “exposure (X)” is a significant predictor of the “mediator (M).” If not, then it is unlikely to mediate anything. Path b: regress “outcome (Y)” on “mediator (M)” to test if the “mediator (M)” is significantly associated with “outcome (Y).” If not, then it is unlikely to mediate anything. Path c: regress “outcome (Y)” on “exposure (X)” to test if the “exposure (X)” is a significant predictor of “outcome (Y)” (total effect). Path c': regress “outcome (Y)” on both “exposure (X)” and “mediator (M)” to test if “mediator (M)” is a significant predictor of “outcome (Y)” and to observe whether the association between “exposure (X)” and “outcome (Y)” is attenuated when the “mediator (M)” is included (direct effect).

We then assessed whether BAA mediates the association between periodontal disease and cognitive function. We performed formal mediation analyses following Baron and Kenny’s framework.^36^ The conceptual mediation model is illustrated in Figure 1, with paths labeled as follows: path a = effect of periodontal condition on BAA (exposure→mediator), path b = effect of BAA on cognitive score (mediator→outcome), path c = total effect of periodontal condition on cognitive score (without mediator), and path c’= direct effect of periodontal condition on cognitive score (with mediator in the model). We carried out separate mediation analyses for each combination of periodontal exposure (periodontitis yes/no, mean CAL, mean PD) and each BAA measure as mediators. All mediation models were adjusted for the full set of covariates. To estimate the indirect (mediated) effect, we used the product-of-coefficients approach and performed bootstrapping with 1000 resamples to generate bias-corrected 95% confidence intervals.^37^ Significance of mediation was determined by the 95% bootstrap interval not including zero (equivalent to the Sobel test). We also computed the proportion of the total effect mediated by BAA using the formula: (β_total effect_ − β_direct effect_)/β_total effect_ × 100%.^22^

For secondary analyses, we examined periodontitis severity in relation to the outcomes. We included indicator variables for moderate and severe periodontitis (reference: no/mild) in regression models for DSST and BAA outcomes and tested for a linear trend by modeling severity as an ordinal variable (0 = no/mild, 1 = moderate, 2 = severe). These models used the same covariates as the main models, allowing assessment of a dose-response by disease severity. The results of the mediation effects by periodontitis severity are presented in Table S4.

## Results

### Participant characteristics

Table 1 presents the characteristics of the 1950 participants, stratified by periodontitis status (no, mild, moderate, severe). The mean age was 70.5 years (SD 7.7). Overall, 59.9% of the sample had periodontitis (19.8% mild, 28.8% moderate, 11.3% severe), while 40.1% had no periodontitis. Participants with periodontitis—especially those with moderate or severe disease—tended to be slightly older and more likely to be male, non-White, have lower education and income, be current smokers, and have cardiovascular disease and hypertension, compared with those without periodontitis (all *P* < .05). Additionally, both BAAs were highly correlated with the other (Pearson correlation *r* = 0.84) and with chronological age (Figure S2, see online supplementary material for a color version of this figure). Biological age estimates increased with periodontal disease severity. For example, the mean KDM-estimated age was 54.5 years in participants without periodontitis vs. 58.6 years in those with severe disease, and the mean PhenoAge was 65.1 vs. 70.4 years, respectively. Consistently, median KDMAge Acceleration ranged from approximately −1.3 years (no disease) to +0.8 years (severe), with a similar gradient for PhenoAge Acceleration (trend *P* < .001 for both). Cognitive performance also worsened with increasing periodontitis severity: the mean DSST score was 46.8 in those with no periodontitis vs. 35.9 in those with severe periodontitis (*P* < .001 across groups).

**Table 1.** Characteristics among older adults aged ≥60 stratified by severity of periodontitis: NHANES 1999-2002 (*N* = 1950).^a^

Characteristics	Total	No periodontitis	Mild periodontitis	Moderate periodontitis	Severe periodontitis	*P* ^b^
***N* (%)**	1950	781 (40.1)	387 (19.8)	562 (28.8)	220 (11.3)	
**Chronological age (years), *M* (SD)**	70.53 (7.67)	70.36 (7.72)	70.49 (7.48)	70.15 (7.63)	72.16 (7.76)	.008
**Men, *n* (%)**	971 (49.8)	333 (42.6)	210 (54.3)	331 (58.9)	97 (44.1)	<.001
**Race/ethnicity, *n* (%)**						.008
** Non-Hispanic White**	1161 (59.5)	506 (64.8)	226 (58.4)	308 (54.8)	121 (55.0)	
** Non-Hispanic Black**	260 (13.3)	86 (11.0)	48 (12.4)	94 (16.7)	32 (14.5)	
** Hispanics**	490 (25.1)	173 (22.2)	103 (26.6)	149 (26.5)	65 (29.5)	
** Others**	39 (2.0)	16 (2.0)	10 (2.6)	11 (2.0)	2 (0.9)	
**Education, *n* (%)**						<.001
** Less than high school**	707 (36.3)	222 (28.4)	137 (35.4)	232 (41.3)	116 (52.7)	
** High school graduate**	454 (23.3)	215 (27.5)	76 (19.6)	106 (18.9)	57 (25.9)	
** Some college**	416 (21.3)	176 (22.5)	106 (27.4)	103 (18.3)	31 (14.1)	
** Bachelor’s degree or higher**	373 (19.1)	168 (21.5)	68 (17.6)	121 (21.5)	16 (7.3)	
**Annual household income, *n* (%)**						<.001
** <$20 000**	817 (41.9)	310 (39.7)	150 (38.8)	236 (42.0)	121 (55.0)	
** $20 000-$75 000**	923 (47.3)	380 (48.7)	196 (50.6)	254 (45.2)	93 (42.3)	
** >$75 000**	210 (10.8)	91 (11.7)	41 (10.6)	72 (12.8)	6 (2.7)	
**Smoking status, *n* (%)**						.002
** Non-smoker**	960 (49.2)	418 (53.5)	184 (47.5)	262 (46.6)	96 (43.6)	
** Former smoker**	792 (40.6)	307 (39.3)	165 (42.6)	225 (40.0)	95 (43.2)	
** Current smoker**	198 (10.2)	56 (7.2)	38 (9.8)	75 (13.3)	29 (13.2)	
**Alcohol intake ≥12 drinks per year, *n* (%)**	1200 (61.5)	448 (57.4)	248 (64.1)	360 (64.1)	144 (65.5)	.021
**Time since last dental visit, *n* (%)**						<.001
** <1 year**	1256 (64.4)	571 (73.1)	244 (63.0)	333 (59.3)	108 (49.1)	
** 1-3 years**	322 (16.5)	106 (13.6)	70 (18.1)	103 (18.3)	43 (19.5)	
** >3 years**	372 (19.1)	104 (13.3)	73 (18.9)	126 (22.4)	69 (31.4)	
**Body mass index, *M* (SD)**	28.37 (5.36)	27.96 (5.11)	28.07 (5.26)	28.75 (5.41)	29.49 (6.18)	<.001
**Chronic conditions, *n* (%)**						
** Hypertension**	970 (49.7)	385 (49.3)	178 (46.0)	283 (50.4)	124 (56.4)	.103
** Diabetes mellitus**	334 (17.1)	113 (14.5)	74 (19.1)	105 (18.7)	42 (19.1)	.089
** Arthritis**	889 (45.6)	365 (46.7)	164 (42.4)	249 (44.3)	111 (50.5)	.213
** Dyslipidemia**	808 (41.4)	372 (47.6)	151 (39.0)	206 (36.7)	79 (35.9)	<.001
** Cardiovascular disease**	389 (19.9)	150 (19.2)	58 (15.0)	122 (21.7)	59 (26.8)	.003
**Periodontal condition, *M* (SD)**						
** Mean CAL (mm) **	1.62 (1.34)	0.74 (0.43)	1.33 (0.56)	2.06 (0.82)	3.42 (1.99)	<.001
** Mean PD (mm)**	1.38 (0.59)	1.20 (0.42)	1.34 (0.51)	1.47 (0.48)	1.96 (0.82)	<.001
**Biological age**						
** KDMAge (years), *M* (SD)**	55.57 (9.72)	54.54 (9.59)	55.25 (9.40)	56.02 (9.80)	58.63 (9.93)	<.001
** KDMAge Acceleration, median (IQR)^c^ ^,^ ^d^**	-0.58 (-3.45, 2.48)	-1.29 (-3.81, 1.41)	-0.67 (-3.60, 2.08)	0.25 (-2.75, 3.23)	0.75 (-2.56, 4.01)	<.001
** PhenoAge (years), *M* (SD)**	66.47 (10.46)	65.14 (10.21)	66.28 (10.47)	66.91 (10.27)	70.39 (10.80)	<.001
** PhenoAge Acceleration, median (IQR)^c^ ^,^ ^d^**	-0.99 (-4.11, 2.74)	-1.85 (-4.81, 1.44)	-1.20 (-4.45, 2.54)	0.03 (-3.28, 3.50)	0.48 (-3.40, 5.91)	<.001
**Cognitive functioning (DSST), *M* (SD)**	43.47 (18.77)	46.77 (18.35)	43.90 (19.55)	41.55 (18.21)	35.92 (17.54)	<.001

Abbreviations: CAL, clinical attachment loss; DSST, digit symbol substitution test; IQR, interquartile range; KDM, Klemera-Doubal Method; NHANES, National Health and Nutrition Examination Survey Study; PD, probing depth; *M*, mean; SD, standard deviation.

a Estimates for means, SD, and proportions (%) were adjusted for sampling weights of NHANES 1999-2002.

b Comparisons were performed using ANOVA for continuous variables and the Chi-squared tests for categorical variables.

c Non-normal distribution continuous variable, median (interquartile range).

d BAA is denoted using residuals from regressing each biological aging measure on chronological age. A positive value indicates an “accelerated” biological aging and a negative value indicates a “decelerated” biological aging.

### Association of periodontitis with cognitive function and BAA

We first examined the direct association between periodontal status and cognitive performance using multivariable linear regression (Table 2, outcome = DSST score). In the fully adjusted model (Model 4), moderate periodontitis was associated with a −0.102 (SE = 0.044, *P* < .05) SD difference in DSST, and severe periodontitis with a −0.165 (SE = 0.061, *P* < .01) SD difference, independent of sociodemographic, behavioral, and health-related factors. The linear trend across severity levels was significant (*P* for trend = .004), reinforcing that as periodontal disease worsens, cognitive performance tends to worsen as well. Treating the periodontal measures as continuous variables yielded consistent results: each 1 mm increment in mean CAL was associated with β = −0.048 (SE 0.016, *P* < .01) for DSST, and each 1 mm higher mean PD with β = −0.125 (0.030, *P* < .001) in DSST (Model 4).

**Table 2.** Associations of periodontal condition with BAA and cognitive functioning (NHANES 1999-2002; *N* = 1950).

Variable	Model 1	Model 2	Model 3	Model 4
***Outcome: Cognitive functioning (DSST score)***				
**Periodontitis (Ref. No)**				
** Mild**	-0.153 (0.061)*	-0.055 (0.049)	-0.050 (0.049)	-0.049 (0.049)
** Moderate**	-0.279 (0.054)**	-0.132 (0.044)***	-0.124 (0.044)***	-0.102 (0.044)*
** Severe**	-0.578 (0.075)**	-0.210 (0.061)**	-0.207 (0.061)**	-0.165 (0.061)***
**Mean CAL (mm)**	-0.160 (0.020)**	-0.153 (0.019)**	-0.042 (0.017)*	-0.048 (0.016)***
**Mean PD (mm)**	-0.295 (0.045)**	-0.328 (0.044)**	-0.148 (0.038)**	-0.125 (0.030)**
***Outcome: KDMAge Acceleration***				
**Periodontitis (Ref. No)**				
** Mild**	0.568 (0.311)	0.247 (0.301)	0.188 (0.299)	0.312 (0.290)
** Moderate**	1.705 (0.277)**	1.211 (0.270)**	1.084 (0.269)**	0.928 (0.261)**
** Severe**	2.135 (0.382)**	2.016 (0.373)**	1.887 (0.372)**	1.456 (0.363)**
**Mean CAL (mm)**	0.493 (0.097)**	0.493 (0.097)**	0.292 (0.097)***	0.221 (0.100)*
**Mean PD (mm)**	1.108 (0.218)**	1.121 (0.219)**	0.633 (0.217)***	0.561 (0.271)***
***Outcome: PhenoAge Acceleration***				
**Periodontitis (Ref. No)**				
** Mild**	1.001 (0.387)***	0.607 (0.375)	0.545 (0.375)	0.516 (0.346)
** Moderate**	1.999 (0.345)**	1.348 (0.336)**	1.226 (0.337)**	0.906 (0.311)***
** Severe**	3.299 (0.476)**	2.992 (0.465)**	2.876 (0.465)**	2.189 (0.432)**
**Mean CAL (mm)**	0.789 (0.120)**	0.784 (0.120)**	0.506 (0.121)**	0.475 (0.124)**
**Mean PD (mm)**	1.620 (0.272)**	1.660 (0.273)**	1.021 (0.270)**	0.967 (0.272)**

Weighted β-coefficients (Standard Errors) are reported. Model 1: unadjusted. Model 2: adjusted for sociodemographic variables (age, sex, race/ethnicity, education, and income). Model 3: additionally adjusted for behavioral variables (smoking, alcohol intake, and dental visit). Model 4: additionally adjusted for health-related variables (body mass index, hypertension, diabetes mellitus, arthritis, dyslipidemia, cardiovascular disease).

Abbreviations: CAL, clinical attachment loss; DSST, Digit Symbol Substitution Test; PD, probing depth.

* *P* < .05.

** *P* < .001.

*** *P* < .01.

We next assessed whether poorer periodontal status is associated with BAA (Table 2, outcomes = BAA measures). In the fully adjusted model (Model 4), participants with moderate periodontitis had an adjusted mean KDMAge Acceleration about 0.928 (SE = 0.261, *P* < .001) years higher than those without periodontitis, and those with severe periodontitis had 1.456 (SE = 0.363, *P* < .001) years higher KDM-BAA, independent of other factors. We observed a similar pattern for PhenoAge Acceleration, though effect sizes were slightly larger. Moderate periodontitis was associated with an adjusted 0.906 (SE = 0.311, *P* < .01) years of PhenoAge Acceleration compared with no disease, and severe periodontitis with 2.189 (SE = 0.432, *P* < .001) years. There was a significant linear trend such that each increase in severity category corresponded to higher BAA (*P* for trend = .01 for PhenoAge, 0.03 for KDM). Continuous measures confirmed this dose-response: each 1 mm higher mean CAL corresponded to +0.221 (0.100) years KDMAge Acceleration (*P* = .03) and +0.475 (0.124) years PhenoAge Acceleration (*P* < .001); each 1 mm higher mean PD corresponded to +0.561 (0.271) years KDMAge Acceleration (*P* < .01) and +0.967 (0.272) years PhenoAge Acceleration (*P* < .001). These results reinforce a dose-response relationship: more severe periodontal condition aligns with progressively greater BAA.

### Association of BAA with cognitive function

We then tested whether higher BAA is associated with worse cognitive performance (Table 3). As expected, each additional year of KDMAge was associated with a decrease of about 0.023 (*P* < .001) SD in DSST in fully adjusted models. More pertinent to our hypothesis, both KDMAge Acceleration and PhenoAge Acceleration showed significant inverse relationships with cognition. In a fully adjusted model, each +1 year of KDMAge Acceleration was associated with a −0.010 (SE = 0.004, *P* = .016) SD decrease in the cognitive score. For PhenoAge Acceleration, each additional year of acceleration was associated with a −0.010 (SE = 0.002, *P* = .002) SD decrease in DSST.

**Table 3.** Associations of BAA and cognitive functioning (NHANES 1999-2002; *N* = 1950).

Variable	Model 1	Model 2	Model 3	Model 4
**KDMAge**	-0.027 (0.002)*	-0.025 (0.002)*	-0.024 (0.002)*	-0.023 (0.002)*
**KDMAge Acceleration**	-0.026 (0.004)*	-0.015 (0.004)*	-0.016 (0.004)*	-0.010 (0.004)**
**PhenoAge**	-0.027 (0.002)*	-0.023 (0.002)*	-0.023 (0.002)*	-0.021 (0.002)*
**PhenoAge Acceleration**	-0.027 (0.003)*	-0.015 (0.002)*	-0.015 (0.002)*	-0.010 (0.002)***

Model 1: unadjusted. Model 2: adjusted for sociodemographic variables (age, sex, race/ethnicity, education, and income). Model 3: additionally adjusted for behavioral variables (smoking, alcohol intake, and dental visit). Model 4: additionally adjusted for health-related variables (body mass index, hypertension, diabetes mellitus, arthritis, dyslipidemia, cardiovascular disease). Weighted β-coefficients (standard errors) are reported.

Abbreviation: KDM, Klemera-Doubal Method.

* *P* < .001.

** *P* < .05.

*** *P* < .01.

### Mediation analysis: BAA as mediators

Given the above associations, we formally tested the mediating role of BAA in the periodontitis-cognition association (Table 4). When considering any periodontitis (yes/no) as the exposure, we found that KDMAge Acceleration mediated a small but significant portion of its association with cognition. Periodontitis was associated with higher KDMAge Acceleration (path a coefficient = 0.812, SE = 0.220, *P* < .001), and higher KDMAge Acceleration was in turn associated with worse cognition (path b = −0.009, SE = 0.004, *P* = .021). The total effect of periodontitis on cognitive score was β_total_ = −0.095 (*P* < .01), and this was reduced to a direct effect (c’) of β = −0.088 (*P* < .05) after accounting for KDMAge Acceleration in the model. The difference corresponds to a statistically significant indirect effect (a * b = −0.007), which amounted to 7.3% of the total association between periodontitis and cognition. In contrast, PhenoAge Acceleration explained about 8.3% of the periodontitis-cognition association, with path a = 1.004 (SE = 0.262, *P* < .001) and path b = −0.008 (SE = 0.003, *P* = .003).

**Table 4.** Mediating role of BAA on the association between periodontitis and cognitive functioning (NHANES 1999-2002; *N* = 1950).^a^^,b^

β-coefficient (SE)	Path a	Path b	Path c’ (direct effect)	Total effect	Proportion mediated (%)
***Exposure: Periodontitis (yes vs. no)***					
**Mediator: KDMAge Acceleration**	0.812 (0.220)*	-0.009 (0.004)**	-0.088 (0.037)**	-0.095 (0.036)***	7.3
**Mediator: PhenoAge Acceleration**	1.004 (0.262)*	-0.008 (0.003)***	-0.087 (0.037)**	-0.095 (0.036)***	8.3
***Exposure: Mean CAL (per 1mm)***					
**Mediator: KDMAge Acceleration**	0.221 (0.100)**	-0.015 (0.005)***	-0.044 (0.015)***	-0.048 (0.017)***	7.0
**Mediator: PhenoAge Acceleration**	0.475 (0.124)*	-0.015 (0.004)*	-0.040 (0.016)**	-0.048 (0.016)***	15.1
***Exposure: Mean PD (per 1mm)***					
**Mediator: KDMAge Acceleration**	0.561 (0.217)***	-0.015 (0.005)***	-0.137 (0.038)*	-0.145 (0.038)*	5.7
**Mediator: PhenoAge Acceleration**	0.967 (0.272)*	-0.015 (0.004)*	-0.131 (0.038)*	-0.145 (0.038)*	9.9

a All models were adjusted for sociodemographic variables (age, sex, race/ethnicity, education, and income), behavioral characteristics (smoking, alcohol intake, and dental visit), and health-related characteristics (body mass index, hypertension, diabetes mellitus, arthritis, dyslipidemia, and cardiovascular disease).

b Analytical steps of mediation model include: path a represents the effect of “exposure” (X) on “mediator (M)”; path b represents the effect of “mediator (M)” on cognitive test scores “outcome” (Y); path c represents the total effect of “exposure” (X) on “outcome” (Y), without the adjustment for “mediator (M)”; and path c’ represents the direct effect of “exposure” (X) on “outcome” (Y), after adjustment for “mediator (M)”; indirect effect represents an effect of “exposure (X)” on “(outcome) Y” mediated by mediator (M). Proportion mediated = (β_total effect_ − β_direct effect_)/β_total effect_ × 100%.

Abbreviations: CAL, clinical attachment loss; KDM, Klemera-Doubal Method; PD, probing depth.

* *P* < .001.

** *P* < .05.

*** *P* < .01.

We also observed significant mediation effects when using the continuous periodontal measures. For mean CAL, the total effect on cognition (per 1 mm) was β_total_ = −0.048 (*P* < .01). Inclusion of KDMAge Acceleration reduced the CAL-cognition coefficient to β_direct effect_ = −0.044 (*P* < .01), corresponding to an indirect effect of −0.004 powered by path a = 0.221 (*P* = .031) and path b = −0.015 (*P* = .003). This indirect effect via KDMAge Acceleration accounted for 7.0% of the association between CAL and cognitive score. Using PhenoAge Acceleration as mediator, the indirect effect was larger: path a = +0.475 (SE = 0.124, *P* < .001) and path b = −0.015 (SE = 0.004, *P* < .001), yielding about 15.1% of the CAL-cognition association mediated by PhenoAge Acceleration. For mean PD, the total effect of PD on DSST was β_total_ = −0.145 per mm (*P* < .001). KDMAge Acceleration explained about 5.7% of this relationship (indirect path a = 0.561, *P* < .01; path b = −0.015, *P* = .005), while PhenoAge Acceleration mediated about 9.9% (path a = 0.967, *P* < .001; path b = −0.015, *P* < .001, Table 4). All mediated pathways described were statistically significant based on bootstrap tests.

## Discussion

In this nationally representative sample of older adults in the United States, periodontal disease was significantly associated with accelerated biological aging and poorer cognitive performance. We included both KDM Biological Age and PhenoAge to capture the concept of biological aging from two angles—one purely algorithmic/phenotypic^19^ and one based on mortality risk prediction.^20^ We found that these measures were highly correlated and yielded consistent results, reinforcing confidence in our findings. Mediation analyses indicated that accelerated biological aging statistically explained part of the periodontitis-cognition link (approximately 5.7%–15.1% of the total effect). In other words, chronic periodontal disease may contribute to cognitive decline partly by hastening systemic biological aging processes. Although the mediated proportion was modest, it was significant, and results were robust across different periodontitis definitions, underscoring the consistency of the relationship. These findings provide first-of-its-kind evidence linking oral health to late-life cognition through measurable aging pathways, extending oral-systemic connections into the realm of geroscience.

Our results extend growing evidence linking poor oral health to cognitive aging. Chronic periodontal infection and inflammation could contribute to systemic inflammation and vascular changes, which in turn may disproportionately affect brain regions responsible for psychomotor speed and executive processes.^38^^,^^39^ Numerous epidemiologic studies have documented that periodontal disease is associated with cognitive impairment and dementia risk in older adults.^3^^,^^9–12^^,^^40^^,^^41^ For example, a recent meta-analysis (46 studies, through 2023) concluded that individuals with periodontal disease had about 25% higher odds of cognitive disorders in cross-sectional analyses and a significantly elevated risk of subsequent cognitive decline and dementia (RR = 1.22 for dementia).^5^ That meta-analysis also found evidence of a stronger association in longitudinal studies of cognitive decline (pooled RR = 3.0).^5^ Another recent systematic review emphasized a bidirectional relationship,^12^ noting that not only can periodontitis contribute to cognitive impairment, but cognitive impairment can worsen oral health, creating a vicious cycle. Our finding that periodontitis correlates with lower DSST scores agrees with these prior observations of worse cognitive performance in those with poor oral health.^3^^,^^9^^,^^33^ Importantly, our study adds evidence of an underlying biological aging process linking oral health and cognition—a connection that has been hypothesized but not previously demonstrated in population studies.

Beyond epidemiologic associations, mechanistic and geroscience-oriented studies provide context for our mediation results. Periodontal disease has been characterized as a contributor to systemic aging, with persistent infection driving inflammatory load that can accelerate aging of the immune and vascular systems.^8^^,^^22^^,^^42^^,^^43^ Animal and cellular studies have long shown that periodontitis can induce oxidative stress and an aged phenotype in distant tissues.^43^ Clinically, each additional missing tooth (often a consequence of severe periodontitis) is associated with a 1.1%–1.4% increase in risk of cognitive impairment and dementia,^44^ highlighting oral health as a contributor to brain aging. Our findings align with these and with another recent study, which found that BAA mediated the periodontitis–cardiovascular disease relationship.^8^ In that study, higher PhenoAge and KDMAge explained part of the excess cardiovascular risk in those with periodontal disease, paralleling our observations with cognitive risk. Together, these studies support a unifying concept: periodontal disease may accelerate fundamental aging pathways,^45^ increasing vulnerability to multiple age-related conditions ranging from heart disease to cognitive decline.

Interpreted through a geroscience lens, our results align with the hypothesis that fundamental aging processes can link disparate age-related conditions.^13^^,^^15^ Geroscience frameworks posit that biological aging—the progressive decline in system integrity—is a common driver of chronic diseases and functional impairments in older adults.^13^^,^^46^ Periodontal disease, traditionally an oral condition, is increasingly recognized as an age-accelerating disease itself.^15^^,^^16^^,^^45^ It is characterized by chronic infection and inflammation of the gums, which can instigate “inflammaging,” the systemic, low-grade inflammation that accompanies aging.^24^^,^^45^ Our finding that periodontitis is linked to elevated biological age markers supports this concept. Notably, the PhenoAge and KDMAge metrics used here have been linked to activation of pro-inflammatory pathways in prior studies,^8^ indicating that higher scores on these indices likely capture an underlying burden of chronic inflammation and immune dysregulation. Thus, it is biologically plausible that long-standing periodontal inflammation accelerates the aging of multiple organ systems (reflected in blood chemistry biomarkers), which in turn contributes to neuronal aging and cognitive decline.

From an oral-systemic aging standpoint, these results extend emerging frameworks that integrate oral health into healthy aging models.^15^^,^^16^ Both periodontitis and late-life cognitive impairment involve heightened systemic inflammation and oxidative stress.^22^^,^^45^ Chronic periodontitis continuously elevates circulating inflammatory markers like IL-6, C-reactive protein, and TNF-α, which are hallmarks of the inflammaging process implicated in neurodegeneration and other age-related pathologies.^17^^,^^30^ These inflammation processes may contribute to cognitive decline by compromising the blood-brain barrier, activating microglia, and promoting the accumulation of neuropathology. Indeed, neuropathological studies have observed that Alzheimer’s disease brains show evidence of chronic inflammation (eg, activated microglia around plaques), and periodontal pathogens such as *Porphyromonas gingivalis* have been linked to increased Alzheimer’s risk via pro-inflammatory effects.^47^ Thus, our findings support a biologically plausible scenario in which an oral inflammatory burden accelerates systemic and brain aging. By demonstrating that physiology-based aging biomarkers partially mediate the oral-cognition link, this study highlights oral health as an integral component of whole-body aging. It identifies accelerated biological aging as a connecting pathway, reinforcing calls to consider oral disease as not only a local pathology but also an upstream contributor to multisystem aging and functional decline.

This study has several limitations that warrant consideration. First, the cross-sectional design limits our ability to draw causal inferences or determine the directionality of associations. It is possible, for example, that individuals with cognitive impairment are less able to maintain oral hygiene, thereby exacerbating periodontitis—a reverse causation that our mediation models cannot fully disentangle. We attempted to mitigate this by adjusting for many confounders. Nonetheless, causality cannot be established in our study, and longitudinal studies are needed. Second, cognitive function was assessed with a single neuropsychological test (DSST), which predominantly measures processing speed and executive function. While DSST is a well-validated indicator of global cognitive aging, it does not capture all cognitive domains (such as memory or visuospatial abilities) that might be affected by oral health.^32^ Future works should examine if periodontitis relates to broader cognitive deficits or clinical dementia diagnoses. Third, our biological aging markers (PhenoAge and KDMAge) are composite indices based on blood chemistry and clinical measures. Although they predict mortality and age-related conditions, they are not direct measures of molecular aging (eg, DNA methylation age). Their accuracy in capturing true biological aging can be limited by their inputs and overlap with inflammatory markers. Some misclassifications in measuring “biological age” are likely, which would bias mediation effects toward null. Moreover, the proportion mediated was relatively small (5%–15%), implying that other pathways (vascular damage, direct neuroinflammation, shared risk factors, etc.) account for most of the periodontitis–cognition link. Fourth, our data were collected in 1999–2002, so the findings may not reflect changes in population health since then. However, we believe the insights remain applicable. The biological links between oral health and systemic aging are not time-dependent, and periodontitis prevalence among older adults remains high today,^28^^,^^29^ while cognitive impairment continues to be a major concern. We specifically chose this dataset for its rich measurements; more recent NHANES cycles did not collect the necessary information concurrently. Replication in contemporary cohorts (or longitudinal cohorts) would help confirm that these associations hold under current conditions, given changes in periodontal treatment and general health over time. Finally, our dataset, while rigorously collected, is now two decades old; changes in population oral health or cognitive risk factors since then are not reflected. However, the fundamental biological relationships are unlikely to have changed, and our use of a nationally representative sample strengthens the generalizability of the findings to older adults in the United States.

## Conclusion

This study provides novel evidence that accelerated biological aging is a mechanistic link between periodontal disease and decline in processing speed/executive function in older adults. Importantly, due to the cross-sectional nature of the data, these results should be interpreted as associations rather than proof of causation. To our knowledge, it is one of the first human studies to demonstrate that an oral health condition can influence late-life cognition through BAA. These findings underscore that the mouth is not isolated from the body’s aging trajectory—chronic oral inflammation can ripple through systemic physiology with consequences for the brain. Thus, maintaining periodontal health may be important not only for preserving teeth but also for promoting healthy aging of the body and mind. Interventions that target oral inflammation and slow biological aging simultaneously could be especially effective in preserving cognitive health. Ultimately, integrating oral health into aging research and care could open new avenues for intervention. Treating gum disease might become part of a multipronged strategy to reduce chronic inflammation, decelerate biological aging, and safeguard cognitive health. Longitudinal and intervention studies are needed to confirm these findings and translate them into actionable strategies for healthy aging.

## Supplementary Material

igaf086_Supplementary_Data

## Data Availability

The data underlying this article are available in the National Health and Nutrition Examination Survey (NHANES) datasets and are publicly available at the National Center for Health Statistics (NCHS) of the U.S. Centers for Disease Control (https://www.cdc.gov/nchs/nhanes/index.html). This study was not preregistered as it represents a secondary analysis of publicly available, de-identified data.
