# Supplementary material for: Accelerated biological aging mediates the association between periodontal disease and cognitive function in older adults
Source: Innov Aging. 2025 Aug 7;9(9):igaf086. doi: 10.1093/geroni/igaf086 (PMC12517742; doi:10.1093/geroni/igaf086)
Supplement: igaf086_Supplementary_Data [file igaf086_supplementary_data.docx]

***Innovation in Aging* Supplementary Material: Qi, Luo, Xu, Liu, & Wu. Accelerated Biological Aging Mediates the Association Between Periodontal Disease and Cognitive Function in Older Adults.**

**Supplementary Figure 1. Flowchart of participants in the National Health and Nutrition Examination Survey Study (NHANES) 1999-2002**

Alt Text: Flowchart showing the selection of a total of 1950 participants who are aged 60 and older, have complete oral health examination, cognitive examination, and lab blood-chemistry data.

**Supplementary Methods. Algorisms for calculating biological age acceleration**

**Klemera-Doubal Method (KDM) biological age:**

KDMAge was based on nine biomarkers (Ln-C-reactive protein (CRP), serum creatinine, glycosylated hemoglobin, serum albumin, serum total cholesterol, serum urea nitrogen, serum alkaline phosphatase, forced expiratory volume, and systolic blood pressure). The values *j* and *i* represent the number of biomarkers and samples, respectively. The values *k*, *q*, and *s* are the regression slope, intercept, and the root means squared error of a biomarker regressed on chronological age, respectively. The value r_j_^2^ represents the variance explained by the regression of chronological age on biomarkers.

$\mathrm{BA}_{E}=\frac{\sum_{j=1}^{m} \left( x_{j}-q_{j} \right)\left( \frac{k_{j}}{s_{j}^{2}} \right)}{\sum_{j=1}^{m} \left( \frac{k_{j}}{s_{j}} \right)^{2}}$ (1)

$r_{char}=\frac{\sum_{j=1}^{m} \frac{r_{j}^{2}}{\sqrt{1-r_{j}^{2}}}}{\sum_{j=1}^{m} \frac{r_{j}}{\sqrt{1-r_{j}^{2}}}}$ (2)

$s_{BA}^{2}=\frac{\sum_{j=1}^{n} \left( \left( \mathrm{BA}_{Ei}-\mathrm{CA}_{i} \right)-\frac{\sum_{i=1}^{n} \left( \mathrm{BA}_{Ei}-\mathrm{CA}_{i} \right)}{n} \right)^{2}}{n}-\left( \frac{1-r_{char}^{2}}{r_{\text{char }}^{2}} \right)\times\left( \frac{\left( \mathrm{CA}_{max}-CA_{min} \right)^{2}}{12m} \right)$ (3)

$\text{KDM-biological age }=\frac{\sum_{j=1}^{m} \left( x_{j}-q_{j} \right)\left( \frac{k_{j}}{s_{j}^{2}} \right)+\frac{\mathrm{CA}}{s_{BA}^{2}}}{\sum_{j=1}^{m} \left( \frac{k_{j}}{s_{j}} \right)^{2}+\frac{1}{s_{\mathrm{BA}}^{2}}}$ (4)

**PhenoAge:**

PhenoAge was calculated by using nine biomarkers, including chronological age, serum albumin, serum creatinine, serum glucose, Ln-CRP, lymphocyte percent, mean cell volume, red blood cell distribution width, serum alkaline phosphatase, and white blood cell count.

*xb* = -19.907 – 0.0336 × Serum Albumin + 0.0095 × Serum Creatinine + 0.1953 × Serum Glucose + 0.0954 × ln (CRP) ­– 0.0120 × Lymphocyte Percent + 0.0268 × Mean Cell Volume + 0.3306 × Red Cell Distribution Width + 0.00188 × Alkaline Phosphatase + 0.0554 × White Blood Cell Count + 0.0804 × Chronological Age

M = 1 – $e^{\left( \frac{-1.51714 \times e^{\left( \mathrm{xb} \right)}}{0.0076927} \right)}$

PhenoAge = 141.50 + $\frac{\ln\left( -0.00553 \times\ln\left( 1 - M \right) \right)}{0.09165}$

**Supplementary Table 1. CDC/AAP case definitions for surveillance of periodontitis**

| **Case** | **Definition** |
| --- | --- |
| No periodontitis | No evidence of mild, moderate, or severe periodontitis |
| Mild periodontitis | ≥2 interproximal sites with attachment loss ≥3mm, and ≥2 interproximal sites with pocket depth ≥4mm (not on same tooth) or one site with pocket depth ≥5mm |
| Moderate periodontitis | ≥2 interproximal sites with attachment loss ≥4mm (not on some tooth), or≥2 interproximal sites with pocket depth ≥5mm (not on same tooth) |
| Severe periodontitis | ≥2 interproximal sites with attachment loss ≥6mm (not on same tooth), and≥1 interproximal sites with pocket depth ≥5mm |

Third molars excluded; total periodontitis is defined as the sum of mild, moderate, and severe disease.

**Reference:**

Eke PI, Page RC, Wei L, Thornton-Evans G, Genco RJ. Update of the case definitions for population-based surveillance of periodontitis. *J Periodontol* 2012;83:1449-1454. <https://doi.org/10.1902/jop.2012.110664>

**Supplementary Table 2. Detailed definitions and categories of chronic conditions**

| **Chronic conditions** | **Measurement, units** | **Categorical definitions** |
| --- | --- | --- |
| Hypertension | Systolic blood pressure (SBP), mm Hg and diastolic blood pressure (DBP), mm Hg; Self-reported physician’s diagnosis | 1. Normal is no self-reported physician’s diagnosis and SBP < 120 mm Hg and DBP < 80 mm Hg; Prehypertension is no self-reported physician’s diagnosis and SBP is 120-140 mm Hg or DBP is 80-90 mm Hg. 2. Hypertension is defined as diagnosed (self-reported) or undiagnosed (no self-reported diagnosis and SBP ≥ 140 mm Hg or DBP ≥ 90 mm Hg) hypertension or currently taking anti-hypertensive medication. |
| Diabetes mellitus | Glycated hemoglobin level, %; Self-reported physician’s diagnosis | 1. Normal is no self-reported physician’s diagnosis and glycated hemoglobin level < 5.7%; Intermediate dysglycemia is no self-reported physician’s diagnosis and glycated hemoglobin level ≥ 5.7%. 2. Diabetes mellitus is defined as self-reported diabetes diagnosis or glycated hemoglobin level of ≥ 5.7%. |
| Arthritis | Self-reported physician’s diagnosis | 1. Normal is no self-reported physician’s diagnosis. 2. Arthritis is self-reported physician’s diagnosis of arthritis. |
| Dyslipidemia | Non-HDL (high-density lipoprotein) cholesterol level, mg/dL; Self-reported physician’s diagnosis | 1. Normal is no self-reported physician’s diagnosis and non-HDL cholesterol level < 130 mg/dL; Intermediate dyslipidemia is no self-reported physician’s diagnosis and non-HDL cholesterol level is 130-160 mg/dL. 2. Dyslipidemia is defined as diagnosed (self-reported) or undiagnosed (no self-reported diagnosis and non-HDL cholesterol level ≥ 160 mg/dL) dyslipidemia or currently taking anti-cholesterolemia medication. |
| Cardiovascular disease | Self-reported physician’s diagnosis | 1. Normal is no self-reported physician’s diagnosis. 2. Cardiovascular disease is self-reported physician’s diagnosis of congestive heart failure or coronary heart disease or angina pectoris or heart attack or stroke. |
| Stroke | Self-reported physician’s diagnosis | 1. Normal is no self-reported physician’s diagnosis. 2. Stroke is self-reported physician’s diagnosis of stroke. |
| Liver disease | Self-reported physician’s diagnosis | 1. Normal is no self-reported physician’s diagnosis. 2. Liver disease is self-reported physician’s diagnosis of any kind of liver condition. |
| Cancer | Self-reported physician’s diagnosis | 1. Normal is no self-reported physician’s diagnosis. 2. Cancer is self-reported physician’s diagnosis of any kind of cancer or malignancy. |

**Supplementary Figure 2. Distributions and correlation matrix of chronological age, biological ages, and corresponding biological age accelerations**

**Supplementary Table 3. Summary of analytical models and variables included**

| **Model (Predictors → Outcome)** | **Exposure/Predictor** | **Mediator (if applicable)** | **Outcome (Dependent Variable)** | **Covariates Included (all models adjust for these)** |
| --- | --- | --- | --- | --- |
| Model 1: Periodontitis → Cognition (total association) | Periodontitis (mild/moderate/severe vs no) | – (no mediator in model) | DSST cognitive score (points) | Age; Sex; Race/Ethnicity; Education; Income; Smoking status; Alcohol intake; Dental visit; Body mass index; Hypertension; Diabetes mellitus; Arthritis; Dyslipidemia; Cardiovascular disease |
| Model 2a: Mediation by KDM BAA | Periodontitis (moderate/severe vs no) | KDM Biological Age Acceleration | DSST cognitive score (points) |  |
| Model 2b: Mediation by PhenoAge BAA | Periodontitis (moderate/severe vs no) | PhenoAge Acceleration | DSST cognitive score (points) |  |
| Model 3: Periodontitis severity (dose-response) | **Periodontitis Severity: no**/mild (ref), moderate, severe | – | DSST cognitive score (points) |  |
| Model 4a: Pocket depth → Cognition (via KDM BAA) | Mean Pocket Depth (per mm, continuous) | KDM Biological Age Acceleration | DSST cognitive score (points) |  |
| Model 4b: Pocket depth → Cognition (via PhenoAge BAA) | Mean Pocket Depth (per mm, continuous) | PhenoAge Acceleration | DSST cognitive score (points) |  |

Notes:

DSST = Digit Symbol Substitution Test; KDM BAA = KDM Biological Age Acceleration; PhenoAge BAA = Phenotypic Age Acceleration.

Each model above was weighted for the NHANES complex survey design. Models 2a/2b and 4a/4b are mediation analyses including the mediator (BAA) in the regression. “Periodontitis (moderate/severe vs no/mild)” is a binary indicator comparing those with moderate or severe periodontitis to those with no or mild periodontitis. Covariates were included as listed; additional covariates (e.g., history of stroke, liver disease, cancer) were tested but not retained in final models if not significant.

**Supplementary Table 4. Mediation of biological age acceleration in the association between periodontitis severity and cognitive functioning (DSST score)**

| **Mediator** | **Exposure (vs No/Mild) – Sample Sizes** | **Path a (β, SE)** | **Path b (β, SE)** | **Direct effect (c’, β, SE)** | **Total effect (c, β, SE)** | **Proportion mediated (%)** |
| --- | --- | --- | --- | --- | --- | --- |
| **KDM Biological Age Acceleration** | **Moderate periodontitis (N = 562) vs No/Mild (N = 1168)** | –0.32 (0.15) | –0.27 (0.10) | –1.16 (0.35) | –1.25 (0.36) | 7.20% |
|  | **Severe periodontitis (N = 220) vs No/Mild (N = 1168)** | –0.73 (0.25) | –0.27 (0.10) | –2.04 (0.50) | –2.23 (0.55) | 8.52% |
| **PhenoAge Acceleration** | **Moderate periodontitis (N = 562) vs No/Mild (N = 1168)** | –0.50 (0.18) | –0.29 (0.07) | –1.10 (0.34) | –1.25 (0.35) | 12.00% |
|  | **Severe periodontitis (N = 220) vs No/Mild (N = 1168)** | –0.90 (0.30) | –0.29 (0.07) | –1.97 (0.53) | –2.23 (0.57) | 11.66% |

Notes:

KDM = Klemera–Doubal Method; BAA = biological age acceleration; DSST = Digit Symbol Substitution Test.

Estimates (Beta coefficients with standard errors) are from mediation models using no or mild periodontitis as the reference group. Results are shown for each periodontitis severity category (moderate, severe) and for two measures of biological age acceleration (KDM Biological Age Acceleration and PhenoAge Acceleration) as the mediator.

Path a = effect of periodontitis severity on the biological age acceleration; Path b = effect of biological age acceleration on DSST score; Direct effect (c’) = direct effect of periodontitis on DSST (adjusted for mediator); Total effect (c) = total effect of periodontitis on DSST (without mediator); Proportion mediated = (c – c’)/c × 100%. Two decimal places are shown throughout, and sample sizes for each group are indicated in parentheses (N). All models are adjusted for covariates and weighted for the NHANES complex survey design.
